# Overexpression of RBM15 modulated the effect of trophoblast cells by promoting the binding ability between YTHDF2 and the CD82 3′UTR to decrease the expression of CD82

**DOI:** 10.1016/j.heliyon.2024.e30702

**Published:** 2024-05-03

**Authors:** Guangning You, Zhe Li, Ling Li, Chengfang Xu

**Affiliations:** Department of Gynecology and Obstetrics, The Third Affiliated Hospital of Sun Yat-sen University, Guangzhou, Guangdong Province, PR China

## Abstract

**Background:**

Pre-eclampsia (PE) is a syndrome with no specific pathological mechanism and is specific to pregnancy. The combined analysis of proteomics and transcriptomics possesses many benefits for treating this disease. m^6^A modification plays a major role in PE; however, mechanism have not been studied clearly. This study investigated the potential mechanism underlying the role of m^6^A in PE.

**Methods:**

Mass spectrometry-based label-free quantitative proteomics and transcriptomics experiments were conducted on the placenta of patients with pre-eclampsia and normal pregnancies, and the two omics were followed by joint analysis. Total m6A modification in placental tissues, HTR8/SVneo cells, and JEG-3 cells was measured by dot blot. The levels of RBM15 and CD82 in tissues and cells were detected using qPCR. The protein levels of G3BP1, RBM15, MMP-2, YTHDF2, and MMP-9 were measured by western blotting. The function, migration, and invasion characteristics of HTR8/SVneo and JEG-3 cells were measured using Transwell assays. SRAMP predicted the m6A modification site in the CD82 mRNA 3′UTR, and this was confirmed using luciferase activity and YTHDF2-RIP.

**Results:**

m^6^A modification was promoted in the PE group, and the RBM15 abundance was increased. Overexpression of RBM15 increased m^6^A modification. However, overexpression of RBM15 suppressed the expression of MMP-2 and MMP-9 and also the migratory and invasive capabilities of HTR8/SVneo and JEG-3 cells. CD82 expression levels were decreased in PE, and CD82 expression was confirmed via qPCR, western blotting and immunofluorescence. Furthermore, RBM15 overexpression reduced CD82 mRNA and protein levels. Luciferase activity and YTHDF2-RIP results verified that overexpression of RBM15 promoted the binding ability between YTHDF2 and the CD82 3′UTR, thereby decreasing CD82 expression. Finally, CD82 overexpression reversed the effect of RBM15 overexpression on the expression of MMP-2 and MMP-9 and on the migratory and invasive capabilities of the cells.

**Conclusions:**

Overexpression of RBM15 hindered the migratory and invasive capabilities of trophoblasts, while concurrently enhancing m6A modification. The potential mechanism was that overexpression of RBM15 promoted the binding capability between YTHDF2 and CD82 3′UTR and decrease the expression of CD82. Thus, this study provides a theoretical basis for the treatment of PE.

## 1 Introduction

Pre-eclampsia (PE) causes pregnancy syndrome with characteristics that include burst-onset hypertension (>20 weeks into pregnancy) and other associated complications such as proteinuria, maternal organ dysfunction, and uteroplacental dysfunction [1]. Notably, impaired placental perfusion in PE triggers the release of soluble factors into the bloodstream, which ultimately culminates into multi-organ damage [2]. Therefore, PE is a prominent contributor to maternal and perinatal mortality and morbidity [2]. PE affects a substantial population of over 300 million women and children globally and exacerbates the likelihood of chronic health complications [1]. However, there are no effective treatments with the exception of termination of pregnancy, and the potential pathological mechanism remains unclear [2,3]. A previous study reported that trophoblast cells play a major role in the placenta, as they can invade the decidua to obtain maternal blood supply. Therefore, abnormalities in trophoblast cells lead to the occurrence of PE [4].

N^6^-methyladenosine (m^6^A), the most universal RNA modification, is a major epigenetic modification of RNA [5]. There are 29 m^6^A methylation regulators that have currently been identified, including 10 “writers”, 2 “erasers”, 11 “readers”, and 6 RNA binding proteins (RBP) that are modulated by m^6^A [6]. The “writers” include CBLL1, RBM15, METTL16, METTL3, and KIAA1429. FTO and ALKBH5 are characterized as “erasers”. “Readers” include YTHDC1, YTHDC2, YTHDF3, eIF3, IGF2BP1, and FMR1, and the RBP include HNRNPC, PRRC2A, ELAVL1, and G3BP2. In a previous study, m^6^A modification significantly increased in trophoblast cells in PE compared to that in their normal counterparts [7]. Studies have demonstrated that m^6^A methylation regulators play major roles in trophoblast functions [[8], [9], [10]]. For example, IGF2BP2 promoted the stability of LincRNA01116 via m^6^A modification to increase trophoblast angiogenesis in PE [8]. METTL3 increases the m^6^A modification of circSETD2, leading to the overexpression of circSETD2 and MCL1 and a decrease in trophoblasts [10]. METTL14 increased circPAPPA2 expression, whereas IGF2BP3 decreased circPAPPA2 expression through m^6^A modification to regulate trophoblast invasion [5]. Another study observed that METTL14 expression was elevated in PE tissues. Its expression promoted FOXO3a expression and enhances autophagy and apoptosis but reduced the proliferation and invasion abilities of trophoblast cells [11]. These findings collectively underscore that m^6^A modification plays a major role in the pathological process of PE.

Biological phenomena are complex and variable, and the regulation of gene expression is also complex. Therefore, multi-omics technology is required to obtain comprehensive information. Mass spectrometry-based label-free quantitative proteomics (MSLQP) is an effective tool for determining the differential expression of proteins in various biological samples. It is highly reproducible, rapid, and precise in regard to its quantitative properties [12]. Combining gel- and chromatography-based separation techniques with subsequent mass spectrometry (MS)-based analysis and bioinformatics approaches enables the handling of issues in various fields of medicine and basic science [13]. Transcriptomics is another high-throughput technique that plays an indispensable role in facilitating the transcriptome-wide analysis of transcriptional variations [14]. Correlation analysis between them is an important aspect that cannot be ignored in systems biology research.

To further investigate the function of m^6^A modification in PE, we performed MSLQP validation to identify the m^6^A methylation regulators, that might play a vital role in PE. Transcriptomics experiment was conducted in PE and control tissues, and we identified the differentially expressed gene CD82. Subsequently, combined with proteomic and transcriptomic analyses, potential mRNAs that were regulated by the m6A methylation regulator CD82 were identified. Potential regulatory mechanisms were verified using luciferase activity and YTHDF2-RIP.

## 2 Methods

### 2.1 Collection of placenta tissues

The inclusion criteria for pre-eclampsia (PE group) included new-onset diastolic blood pressure of ≥90 mm Hg and/or systolic blood pressure of ≥140 mm Hg after 20 weeks into the pregnancy and also any one of the following parameters: urinary protein ≥0.3 g/24 h; the ratio of urinary protein and creatinine ≥0.3; random urinary protein ≥ (+); no proteinuria and including any one of the following organ or system involvement: lung, heart, liver, kidney; abnormal changes in the digestive, blood, and nervous systems; and placental-foetal involvement.

The inclusion criteria for the healthy control group (NC group) included normal healthy pregnant women with no proteinuria, normal blood pressure and no pregnancy syndrome. Patients in the PE group were matched with those in the NC group in terms of age, gestational age, and body mass index at a 1:1 ratio. The exclusion criteria were gestational diabetes mellitus, twin pregnancy, renal disease, primary hypertension, hyperthyroidism, hypothyroidism, and acute and chronic hepatitis.

All the samples used in this study were obtained from the Third Affiliated Hospital of Sun Yat-sen University's Obstetrics Specimen Repository. The samples were from women who had been admitted for childbirth, with the collection period from January 1st to December 31st of the year 2022. The umbilical cord was used as the central reference point, and 5 pieces of placental tissue were extracted from the maternal placenta surface at points 2, 4, 6, 8, and 10. The tissue samples were taken approximately 2–3 cm away from the edge to avoid calcification, bleeding, or other complications. Each sample had dimensions of 0.5*0.5*0.5 cm. First, we use pre-cooled physiological saline to flush away the blood on the placenta. Placenta is packed into cryogenic tubes. Next, the cryogenic tubes are quickly frozen in liquid nitrogen, and finally transferred to −80 °C for storage. The entire process is controlled to be completed within 15 min. Seven placental tissues from each PE and NC group were collected separately. We used three placental tissues for MSLQP and transcriptomic analyses. We used seven placental tissue samples from each PE and NC group for follow-up validation. This study was conducted in accordance with the 1964 Declaration of Helsinki and was approved by the Ethics Committee of the Third Affiliated Hospital of Sun Yat-sen University (ethics number II2023-063-01). Additional informed consent was obtained from all individual participants included in this article.

### 2.2 MSLQP

MSLQP was performed according to a previous study with minor revision [15]. Briefly, 100 mg of placental tissue was placed in lysis buffer and homogenised for 20 min using a mechanically operated homogeniser. The sample was then centrifuged to obtain protein supernatants. The protein concentration was quantified using the BCA method. Subsequently, peptide extraction from the proteins was performed, and this was followed by liquid chromatography-mass spectrometry (LC-MS). Comparisons between the PE and NC groups revealed protein expression fitted with a fold change >1.2, and P < 0.05 (*t*-test). GO and KEGG analyses were conducted based on the significantly differentially expressed proteins (DEPs).

### 2.3 Transcriptomics

TRIzol (Magen) was used to isolate RNA from placental tissues. The A260/A280 absorbance ratio was measured using a NanoDrop ND-2000 spectrophotometer, and RIN values were measured using an Agilent Bioanalyzer 4150. The library was prepared using an ABclonal mRNA-seq Lib Prep Kit (ABclonal, Hubei, China). To purify 1 μg of total RNA to convert to mRNA, oligo (dT) magnetic beads were utilised, and this was followed by mRNA fragmentation. Subsequently, the first and second strands of cDNA were synthesised. Synthesised double-stranded cDNA fragments were joined to a splicing sequence. The PCR product was purified, and library quality evaluation was performed using an Agilent Bioanalyzer 4150. The NovaSeq 6000 sequencing platform PE150 was used for sequencing. The data obtained from the Illumina platform were used for bioinformatics analysis. The gene expression between the PE group and NC group fulfilled the following criteria that included fold change (FC) > 1.5 and P < 0.05 (T-test). Furthermore, Gene Ontology (GO) and Kyoto Encyclopedia of Genes and Genomes (KEGG) analyses were conducted based on the significantly differentially expressed genes.

### 2.4 Combining analysis of MSLQP and transcriptomics analyses

First, we integrated the transcriptome and proteome data. When a protein is identified and expressed at the transcriptome level, genes and proteins are considered to be related. Subsequently, we performed statistical analysis on the quantitative relationships between related proteins and genes while considering both quantifiable and significant differences. We drew a scatter map and calculated the correlation coefficients for significantly correlated proteins and genes. Furthermore, cluster analysis was conducted to assess the mRNA and protein expression patterns more intuitively. These related genes and proteins were also used for GO and KEGG analyses.

### 2.5 Dot blot

Total RNA was isolated using TRIzol reagent. The RNA was diluted to the indicated concentration, and the film was incubated with anti-m6A antibody (proteintech, #68055-1-Ig) at 4 °C. The nylon film was washed three times with TBST (5 min each). The nylon films were then incubated with sheep anti-rabbit IgG-HRP for 1 h. The nylon film was washed an additional three times for 10 min each time. Finally, ECL was loaded onto the nylon film and incubated in the dark for 5 min. Photographs were captured by a developer. The nylon film was placed in 10 ml methylene blue staining buffer for 30 min and then washed for approximately 30–60 s with double distilled water until the background was roughly clean. Images after methylene blue staining were collected using white-light imaging as a load control.

### 2.6 Cell culture and transfection

The supplier of HTR8/SVneo is Guangzhou Kelojie Biotechnology Co., Ltd., and the source of HTR8/SVneo is human placenta. HTR8/SVneo cells (KC4027; Kinlogic) were maintained in RPMI 1640 (Kinlogix; M1002) supplemented with 5 % foetal bovine serum (FBS). The supplier of JEG-3 is Guangzhou Kelojie Biotechnology Co., Ltd., and the source of JEG-3 is human bladder. JEG-3 cells (KC0901; Kinlogic) were cultured in MEM (Kinlogix; M1003) supplemented with 10 % FBS. The supplier of 293T is Guangzhou Kelojie Biotechnology Co., Ltd., and the source of 293T is Human kidney. The 293T cells (Kinlogix; KC4003) were grown in DMEM (high glucose) (Kinlogix; M1001) supplemented with 10 % FBS. The cells were amplified in a humidified incubator (37 °C and 5 % CO2). Lipofectamine 3000 (Thermo Fisher Scientific, #L3000015) was used for transfection according to the manufacturer's instructions.

### 2.7 Western blotting

Proteins were obtained from cells or tissues using RIPA lysis buffer. Protein concentration was measured using a BCA kit. Then, 25 μg of protein per sample was separated by SDS-PAGE., Protein was transferred to PVDF membranes. The membrane was blocked with 5 % non-fat milk for 1 h, incubated with the primary antibody overnight, and then incubated with the secondary antibody for 1 h. Finally, the membrane was incubated with ECL to observe the protein bands. The primary antibodies and secondary antibodies included UltraPolymer Goat anti-Mouse IgG (H&L)-HRP (proteintech, #PR30012), G3BP1 (proteintech, #13057-2-AP), RBM15 (proteintech, #10587-1-AP), YTHDF2 (proteintech, #24744-1-AP), MMP2 (proteintech, #10373-2-AP), NOTUM (proteintech, #14663-1-AP), CD82 (proteintech, #66803-1-Ig), GAPDH(proteintech, #10494-1-AP), UltraPolymer Goat anti-Rabbit IgG (H&L)-HRP (proteintech, #PR30011), and MMP9 (N-terminal) (proteintech, #10375-2-AP).

### 2.8 Construction of recombinant plasmid

Overexpression plasmids (pCDH-RBM15 and pCDH-CD82) and their control plasmids (pCDH-NC) were provided by Jiangsu Genecefe Biotechnology Co., Ltd. The CDS region of RBM15 or CD82 was inserted into the pCDH-CMV-EF1A-EGFP-T2A-Pero vector to construct an overexpressed RBM15 or CD82 vector. The CD82 wild-type fluorescent report plasmid (pmirGLO-WT), mutant plasmid (pmirGLO-mut), and control plasmid (pmirGLO) were provided by Jiangsu Genecefe Biotechnology Co., Ltd.

### 2.9 Detection of the function of migration and invasion

For invasion, Matrigel was dissolved overnight at 4 °C and diluted at a ratio of 1:3 with culture medium. Matrigel (40 μL) was added to the chamber of a precooled plate, and the Transwell plate was incubated at 37 °C for 2 h to coagulate the Matrigel. Basal medium was added into the upper chamber (100 μL) and lower chamber (600 μL), incubated overnight at 37 °C, and removed the next day. The subsequent protocol was the same as that used for the invasion and migration assays. First, we added 1 × 10^5^ cells in 100 μL of basal medium/ml into the upper chamber. The medium with 5 % foetal bovine serum (600 μL) was added into the lower chamber. After culturing in a humidified incubator containing 5 % CO2 for 48 h, the upper chamber was removed, and the cells were wiped using a cotton swab. Migrating and invading cells were fixed with 4 % paraformaldehyde for 15 min. After washing with phosphate buffered saline (PBS), the cells were stained with 1 % crystal violet and photographed under a microscope.

### 2.10 qPCR

To detect mRNA expression, qPCR was performed according to a previous study with a some revisions [16]. Briefly, total RNA was isolated using TRIzol reagent and reverse transcribed into cDNA using the Prime Script RT reagent kit (Roche Life Science, Mannheim, Germany). qPCR was performed using an ABI7500. The primer sequence were showed as follows (5′-3′): RBM15-F: CGAGATAGGAAGCACCGGAC, RBM15-R:CCCCATCCTGTTTCTGGGAC; CD82-F:TCTCTGTCCTGCAAACCTCC, CD82-R:CCTGGGCAATGAGGATCAGG; GAPDH-F:GAGTCAACGGATTTGGTCGT, GAPDH-R:GACAAGCTTCCCGTTCTCAG. NOTUM-F: TACCTGAAGGAGTCCAGGGG; NOTUM-R: CGTTCCACCAGTAGGGGTTC.

### 2.11 Luciferase activity

After transfection for the indicated time, a TransDetect® Double-Luciferase Reporter Assay Kit (Tansgen, #FR201-01) was used to detect the luciferase activity according to the kit protocol.

### 2.12 Immunofluorescence evaluated CD82 expression

CD82 (proteintech, #66803-1-Ig) was used in the immunofluorescence. Paraffin sections were routine dewaxing and hydration (xylene for 10 min, xylene for 10 min, 100 % ethanol for 10 min, 95 % ethanol for 5 min, 70 % ethanol for 5 min, PBS for 5 min, and PBS for 5 min. Then thermal repair was performed (slices was boiled in 0.01 M sodium citrate buffer solution (pH 6.0) for 15 min). After washing with PBS for 5 min, 5 % normal goat serum was added on the slices and incubation at room temperature for 1 h. Then diluted primary antibody was added and incubated at 4 °C overnight. After washing with PBS thrice (2 min each time), fluorescent labeled secondary antibody was added and incubated at room temperature in the dark for 1 h. Washed with PBS thrice (2 min each time), slices incubated with the Hoechst at room temperature for 10 min. Washed with PBS thrice (2 min each time), slices were filmed with mounting medium, and the fluorescence was observed under a fluorescence microscope.

### 2.13 YTHDF2-RIP

A Magna RIP RNA-Binding Protein Immunisation Kit (Millipore, #17-700) was used to conduct YTHDF2-RIP experiments. Briefly, cells were lysed using RIP. YTHDF2 was incubated with magnetic beads to obtain immunoprecipitation magnetic beads that were then incubated with cell lysate to carry out the immunoprecipitation of the RNA-binding protein-RNA complex, and this was followed by the RNA elution from the complex. An RT kit (#R323-01; Vazyme) was used for cDNA synthesis. A SYBR qPCR kit (#Q711-02; Vazyme) was used for qPCR. Relative expression of CD82 was calculated using the 2^-△△Ct^ method. The primers for CD82 were: (5′-3′): CD82 3‘UTR-F:GCGATCTCTCCTGGCCTATC, CD82 3‘UTR-R:GGTAGAAGTCCAGCCCACCT.

### 2.14 m^6^A modification prediction

SRAMP predicted the m^6^A modification site of the CD82 mRNA 3'UTR.

### 2.15 Statistical analysis

Data are presented as mean ± standard deviation. Statistical analysis was performed using SPSS and employed Student's t-test for comparisons between two groups and analysis of variance (ANOVA) for comparisons among more than two groups. *P*-values of less than 0.05 were considered statistically significant. GraphPad Prism software (version 7.0; La Jolla, California, USA) was used to plot data. Data Size of our manuscript is small. Base on previous study [17], the parametric tests were often used for small numbers of measurements as most continuous data satisfied with normality and equal variance.

## 3 Results

### 3.1 Proteins detected by MSLQP in the placenta of pre-eclampsia and normal pregnancy

The general clinical data of pregnant women in the PE group and NC group are presented in Table S1. There were no significant differences observed in maternal age, body mass index, gestational age at delivery, gravidity, parity, placental weight, and newborn weight (P > 0.05). In the PE group, the average systolic blood pressure was 154.00 ± 15.11 mmHg and the average diastolic blood pressure was 97.43 ± 8.75 mmHg. Conversely, the mean systolic blood pressure in the NC group was recorded as 113.71 ± 11.28 mmHg with a mean diastolic blood pressure of 71.29 ± 7 0.78 mmHg (P < 0.0001).

**Fig. 1:**
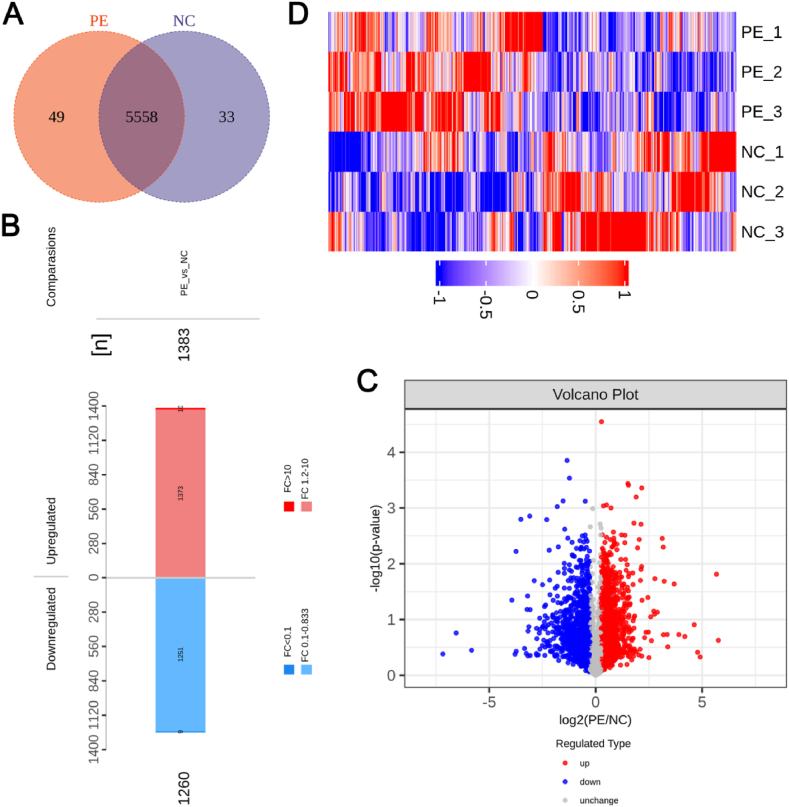
Proteins detected by MSLQP in pre-eclampsia and normal pregnancy placenta. (A) A Venn diagram was used to analyse the number of proteins detected in pre-eclampsia and normal pregnancy placenta. N (biological replicates) = 3 each group. (B) The number of significantly differential proteins between pre-eclampsia and normal pregnancy placenta. Red indicates the up-regulated proteins, and blue indicates the down-regulated proteins. (C) A Volcano plot was used to analyse the proteins detected in the placenta from pre-eclampsia and normal pregnancy. Blue indicated the down-regulated proteins, grey indicated the proteins with no difference in expression between the two groups, and red indicated the up-regulated proteins. (D) A heat map was used to identify significantly differentially expressed proteins between pre-eclampsia and normal pregnancy placenta. Red indicates the up-regulated proteins, and blue indicates the down-regulated proteins. PE, pre-eclampsia; NC, normal pregnancy placenta; FC, fold change.

We identified 5,591 proteins in the NC group and 5,607 proteins in the PE group, and of these, 5,558 were present in the PE and NC groups (Fig. 1A). For filtering significantly differentially expressed proteins, the criteria were fold change (FC) > 1.2 and P < 0.05 (T-test). There were 1,260 proteins that were upregulated and 1,383 proteins that were downregulated in the PE group compared to levels in the NC group (Fig. 1B). The volcano plot and heat map indicate significant differences between the NC and PE groups (Fig. 1C and D). Differentially expressed proteins were well distinguished between the NC and PE groups.

### 3.2 GO and KEGG analysis for the significantly differentially expressed proteins

**Fig. 2:**
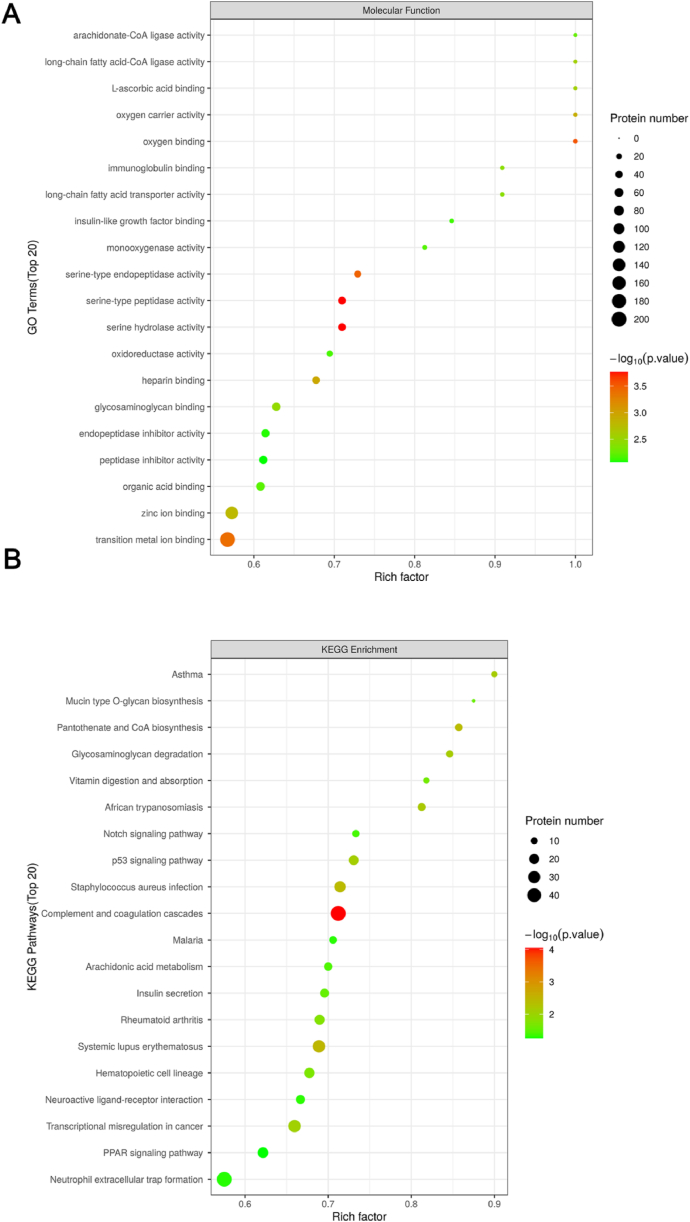
Gene Ontology and Kyoto Encyclopedia of Genes and Genomes were used to analyse the function and signalling pathways of differentially expressed proteins between pre-eclampsia and normal pregnancy placenta. (A) Bubble chart of the top 20 molecular functions after Gene analysis using Blast2Go. (B) Bubble chart of the top 20 signalling pathways as analysed using the Kyoto Encyclopedia of Genes and Genomes.

The top 20 functions were arachidonate-CoA ligase activity, oxygen carrier activity, long-chain fatty acid-CoA ligase activity, immunoglobulin binding, insulin-like growth factor binding, monooxygenase activity, serine hydrolase activity, oxidoreductase activity, heparin binding, glycosaminoglycan binding, peptidase inhibitor activity, and endopeptidase inhibitor activity (Fig. 2A and Table S2). The top 20 pathways were mucin-type O-glycan biosynthesis, pantothenate and CoA biosynthesis, glycosaminoglycan degradation, vitamin digestion and absorption, Notch and p53 signalling pathways, complement and coagulation cascades, insulin secretion, haematopoietic cell lineage, and neutrophil extracellular trap formation (Fig. 2B and Table S3).

### 3.3 Verification of the expression levels of G3BP1, RBM15, and YTHDF2 in placenta

**Fig. 3:**
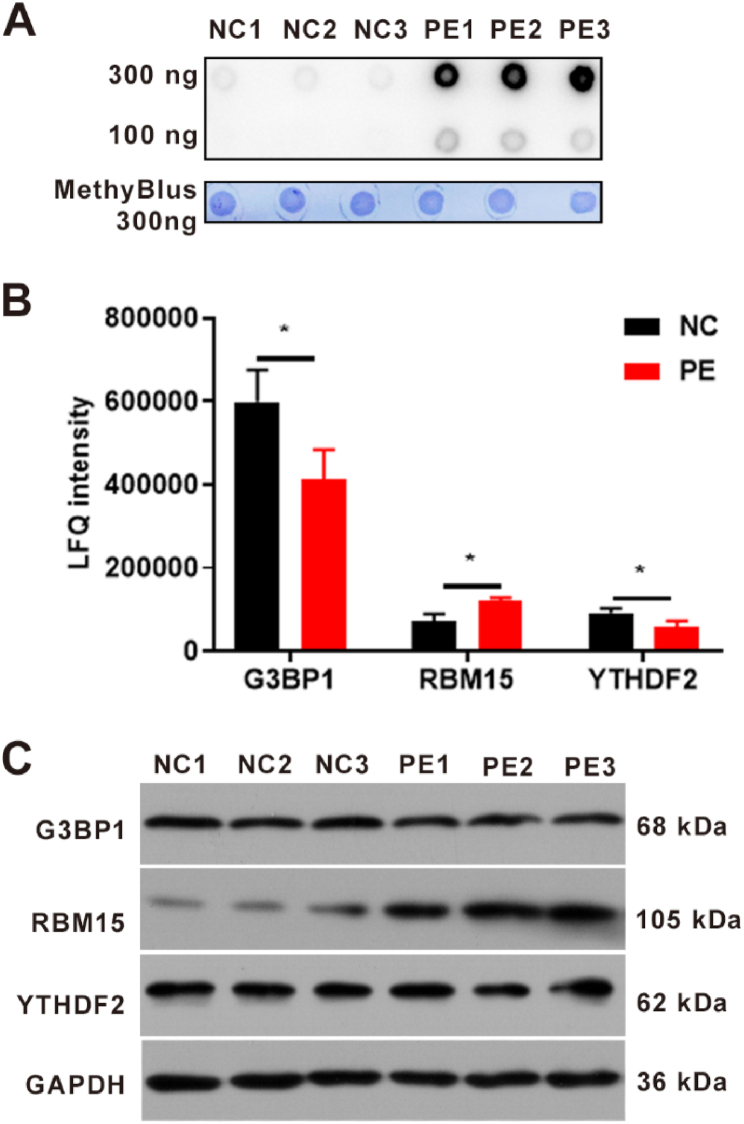
Verification of the expression of m^6^A modification-related proteins. (A) Dot Blot detection of m^6^A modification of total RNA in pre-eclampsia and normal pregnancy placenta. N (biological replicates) = 3 each group. (B) Proteins detected by MSLQP in the placenta of pre-eclampsia and normal pregnancy revealed that there were significant differences in m^6^A modification-related protein expression levels, including G3BP1, RBM15, and YTHDF2. N (biological replicates) = 3 each group. (C) Western blotting was used to detect the expression of G3BP1, RBM15, and YTHDF2 in placenta from pre-eclampsia and normal pregnancy. N (biological replicates) = 3 each group. *, p value less than 0.05. PE, pre-eclampsia; NC, normal pregnancy placenta; G3BP1, Ras GTPase-activating protein-binding protein 1; RBM15, RNA-binding protein 15; YTHDF2, YTH domain-containing family protein 2; MSLQP: mass spectrometry-based label-free quantitative proteomics.

m^6^A modification plays a major role in the placenta, and the present study primarily focused on G3BP1, RBM15, and YTHDF2 proteins [5,7,18]. First, the Dot Blot test revealed the m^6^A modification levels of total RNA in the NC and PE groups. The results indicated that levels of m^6^A modification were dramatically enhanced in the PE group compared to those in the NC group (Fig. 3A). According to the MSLQP results, there were 16 m^6^A modification proteins (Table 1). Among the top three differentially expressed proteins, G3BP1, YTHDF2, and RBM15 expression was increased in the PE group (Fig. 3B and Table 1). Western blot results revealed no change in the expression of G3BP1 and YTHDF2 between the PE and NC groups; however, the expression levels of RBM15 were higher in the PE group than they were in the NC group (Fig. 3C). Therefore, RBM15 was selected for further analysis.

**Table 1:** Expression of all proteins related to m^6^A modification according to mass spectrometry-based label-free quantitative proteomics.

Protein	Protein Name	Gene Name	PE/NC	*p* value
**Q96T37**	RNA-binding protein 15	RBM15	1.671618	0.013053
**Q9Y5A9**	YTH domain-containing family protein 2	YTHDF2	0.646685	0.02925
**Q13283**	Ras GTPase-activating protein-binding protein 1	G3BP1	0.691837	0.037467
**Q9H4Z3**	mRNA (2-O-methyladenosine-N(6)-)-methyltransferase	PCIF1	1.238051	0.04533
**P22626**	Heterogeneous nuclear ribonucleoproteins A2/B1	HNRNPA2B1	1.178333	0.070218
**Q7Z739**	YTH domain-containing family protein 3	YTHDF3	1.170227	0.16122
**Q15007**	Pre-mRNA-splicing regulator WTAP	WTAP	0.868989	0.349729
**Q9C0B1**	Alpha-ketoglutarate-dependent dioxygenase FTO	FTO	1.138104	0.376069
**P07910**	Heterogeneous nuclear ribonucleoproteins C1/C2	HNRNPC	1.092204	0.377816
**Q14152**	Eukaryotic translation initiation factor 3 subunit A	EIF3A	0.935475	0.39791
**O00425**	Insulin-like growth factor 2 mRNA-binding protein 3	IGF2BP3	1.126944	0.442491
**Q9UN86**	Ras GTPase-activating protein-binding protein 2	G3BP2	0.921911	0.454092
**Q15717**	ELAV-like protein 1	ELAVL1	0.927647	0.530973
**Q96MU7**	YTH domain-containing protein 1	YTHDC1	0.869959	0.633737
**Q9Y6M1**	Insulin-like growth factor 2 mRNA-binding protein 2	IGF2BP2	0.842749	0.804694
**Q06787**	Synaptic functional regulator FMR1	FMR1	1.072123	0.818876

### 3.4 RBM15 inhibited trophoblast cell function and promoted m^6^A modification levels in HTR8/SVneo and JEG-3 cells

**Fig. 4:**
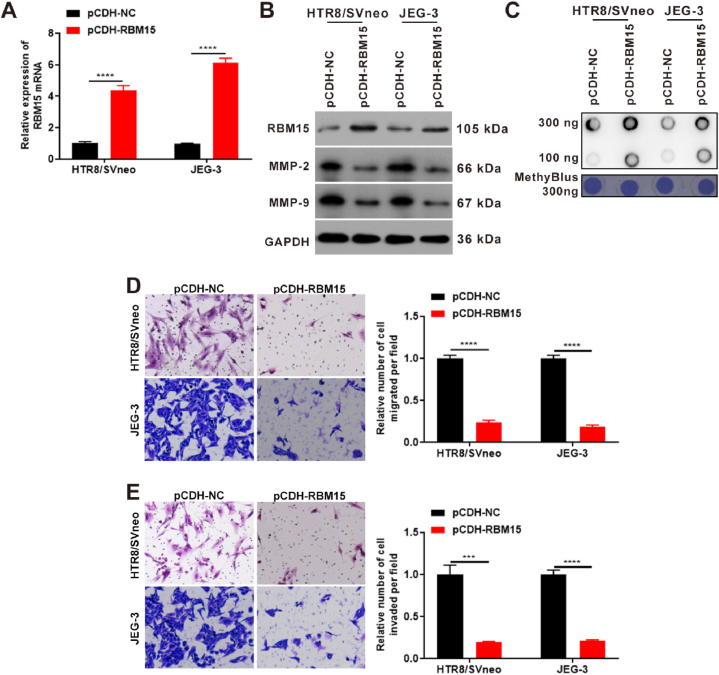
RBM15 inhibited trophoblast cell function and promoted m^6^A modification levels in HTR8/SVneo and JEG-3 cells. (A) qPCR detected the expression of RBM15 mRNA levels after overexpression of RBM15 for 24 h. (B) Western blotting was used to detect the expression levels of RBM15, MMP-2, and MMP-9 after overexpression of RBM15 for 48 h. (C) Dot Blots were used to detect the m^6^A modification of total RNA after overexpression of RBM15 for 24 h in both HTR8/SVneo and JEG-3 cells. (D) Transwell assays were used to assess the changes in cell migration after overexpression of RBM15 for 48 h. (E) Transwell assays were used to detect the changes in cell invasion after overexpression of RBM15 for 48 h ***, p value less than 0.001; ****, p value less than 0.0001. RBM15, RNA-binding protein 15; MMP-2, matrix metallopeptidase 2; MMP-9, matrix metallopeptidase 9; GAPDH, glyceraldehyde-3-phosphate dehydrogenase; pCDH-NC, the vector of pCDH.

In our study, the pCDH-RBM15 vector was constructed. qPCR results revealed that RBM15 mRNA levels increased approximately 4- or 6-fold after transfection with the pCDH-RBM15 vector (Fig. 4A). Western blotting indicated that the RBM15 protein levels increased after transfection with the pCDH-RBM15 vector, thus suggesting that overexpression of RBM15 was successful (Fig. 4B). Dot Blot results demonstrated that m^6^A modification was promoted after RBM1 overexpression (Fig. 4C). Transwell assays indicated that cell migration was decreased to 25 % after RBM15 overexpression (Fig. 4D). Moreover, cell invasion was decreased to 25 % after RBM15 overexpression (Fig. 4E). Additionally, MMP-2 and MMP-9 expression was simultaneously suppressed (Fig. 4B). These results indicate that RBM15 regulates trophoblast function and promotes m^6^A modification.

### 3.5 Transcriptome sequencing in the placenta from pre-eclampsia and normal pregnancy

**Fig. 5:**
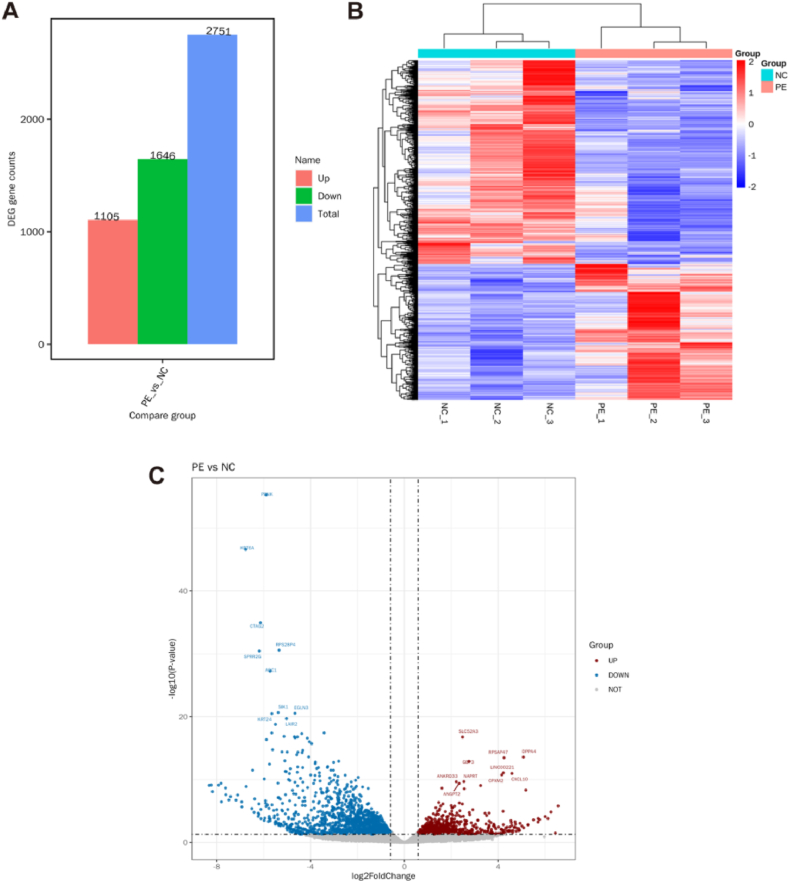
Differentially expressed genes using transcriptome sequencing in pre-eclampsia and normal pregnancy placenta. (A) Number of significantly differential expressed mRNAs in pre-eclampsia and normal pregnancy placenta. Red indicated the number of elevated mRNAs in the PE group, green indicated the number of downregulated mRNAs in the PE group, and blue indicated the number of total significantly differential expressed mRNAs between the two groups. N (biological replicates) = 3 each group. (B) A heat map was used to indicate the significantly differential expressed mRNAs in pre-eclampsia and normal pregnancy placenta. Blue indicated downregulated mRNAs in the PE group, and red indicated upregulated mRNAs in the PE group. N (biological replicates) = 3 each group. (C) A Volcano plot was used to present the detected mRNAs between pre-eclampsia and normal pregnancy placenta. Blue indicated downregulated mRNAs in the PE group, red indicated upregulated mRNAs in the PE group, and grey indicated no significantly expressed mRNAs between PE and NC group. N (biological replicates) = 3 each group. PE, pre-eclampsia; NC, normal pregnancy placenta; DEGs: differentially expressed genes.

Transcriptome sequencing was performed to identify the differentially expressed mRNAs between the NC and PE groups. In the screening of significantly different mRNAs, fold change (FC) > 1.5 and P < 0.05 were used as the criteria, and a total of 2,751 mRNAs were obtained. Of these, 1,105 were upregulated and 1,646 were downregulated in the PE group compared to levels in the NC group (Fig. 5A). We also used a heat map and volcano plot to present the significantly differentially expressed mRNAs between PE and normal pregnancy placenta (Fig. 5B and C). Differentially expressed mRNAs were also identified between the NC and PE groups.

### 3.6 The functions and signalling pathway

**Fig. 6:**
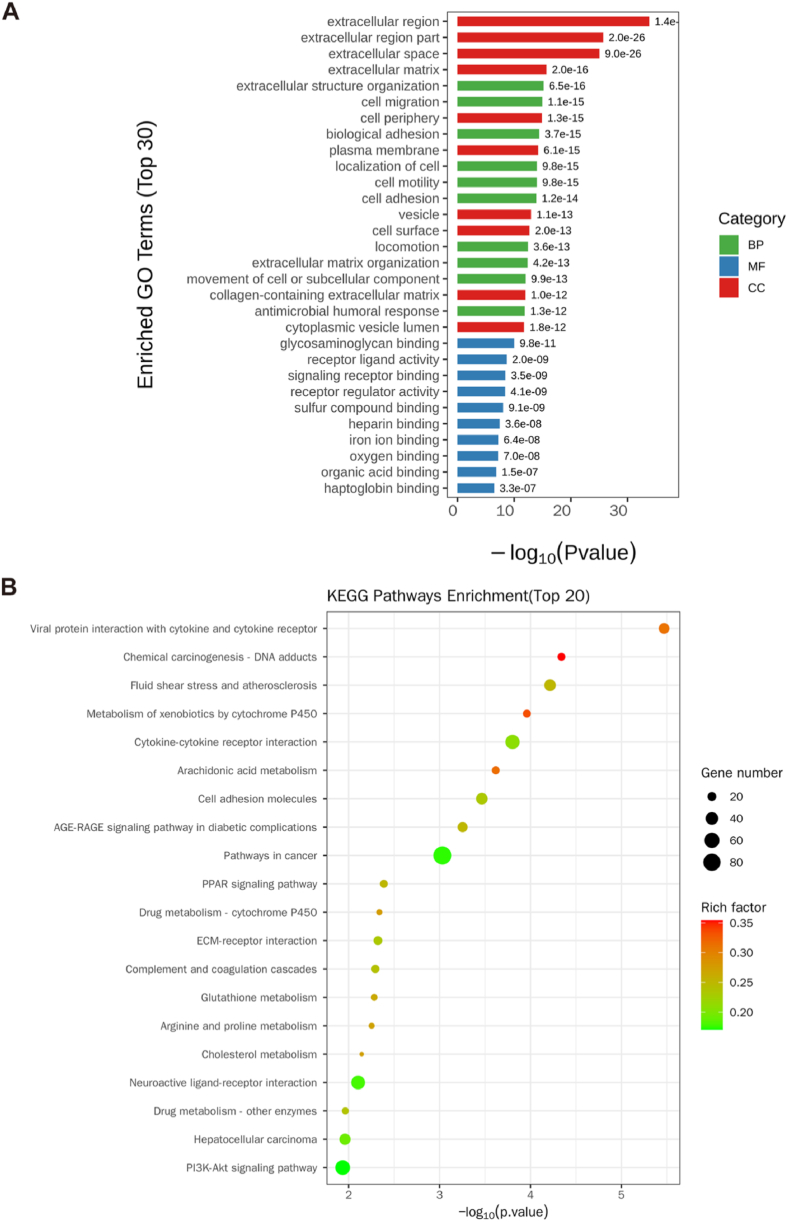
Gene Ontology and Kyoto Encyclopedia of Genes and Genomes were used to analyse the function and signalling pathways of differentially expressed mRNAs between pre-eclampsia and normal pregnancy placenta. (A) Column chart of the top 30 functions after analysis by Gene Ontology using Blast2Go (https://www.blast2go.com/). (B) Bubble chart of the top 20 signal pathways as analysed by the Kyoto Encyclopedia of Genes and Genomes.

The top 30 GO terms included extracellular region part; extracellular space; extracellular region; extracellular structure organization; cell migration; cell periphery; biological adhesion; plasma membrane; localization of cell; cell motility; cell adhesion; vesicle; cell surface; locomotion; extracellular matrix organization; movement of cell or subcellular component; collagen-containing extracellular matrix; antimicrobial humoral response; cytoplasmic vesicle lumen; signaling receptor binding; receptor regulator activity; sulphur compound binding, heparin binding, and heptoglobin binding (Fig. 6A and Table S4). The top 20 KEGG terms included the metabolism of xenobiotics by cytochrome P450, arachidonic acid metabolism, cell adhesion molecules, pathways in cancer, PPAR signalling pathway, complement and coagulation cascades, glutathione metabolism, arginine and proline metabolism, cholesterol metabolism, and neuroactive ligand-receptor interactions (Fig. 6B and Table S5).

### 3.7 Combination analysis transcriptome and MSLQP

**Fig. 7:**
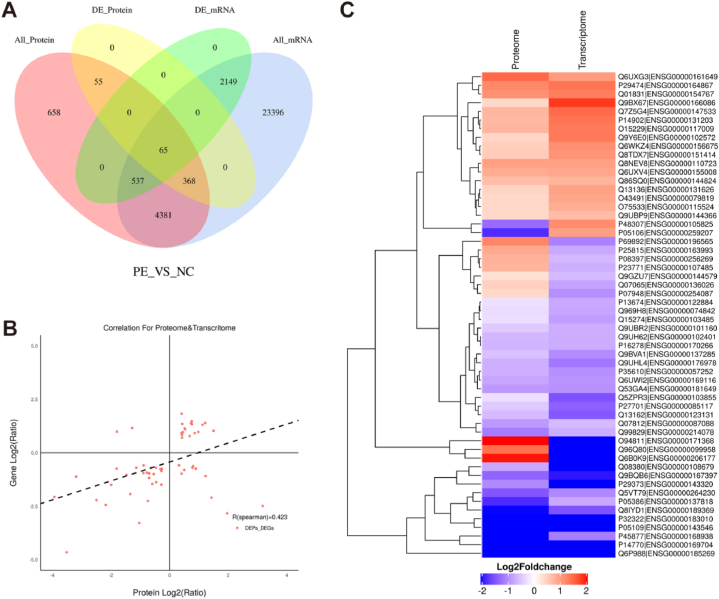
Combination analysis of the transcriptome and mass spectrometry-based label-free quantitative proteomics that was used to assess the transcription and translation characteristics of differentially expressed genes in placenta from pre-eclampsia and normal pregnancy (A) A Venn diagram was used to indicate the number of associations between the transcriptome and proteome at the quantitative and differential expression levels. Red indicates the number of genes corresponding to quantifiable proteins in the NC and PE groups, yellow indicates the number of genes corresponding to significantly differentially expressed proteins, blue indicates the number of genes detected in the NC and PE groups, and green indicates the number of significantly differentially expressed genes. (B) Analysis of correlated genes. (C) Heat map indicating the 56 correlated genes and proteins. The left panel presents the proteome, the right panel indicates the transcriptome, and each line represents a protein/mRNA. Red indicates upregulation, and blue indicates downregulation. PE, pre-eclampsia; DE, differential expressed.

Combined analysis of the transcriptome and MSLQP was performed as follows. First, for the quantifiable 6,064 proteins detected in the PE and NC groups using MSLQP and quantifiable 30,896 mRNAs detected using transcriptome analysis, there were 5,351 correlated genes. A total of 488 genes corresponded to significantly differentially expressed proteins, and there were 2,751 significantly differentially expressed genes, where the number of correlated genes was 65 (Fig. 7A). Correlation analysis was performed for these 65 correlated genes, and the R (Spearman) value was 0.423, thus indicating a positive correlation (Fig. 7B). Fifty-six correlated genes are presented in Fig. 7C. Moreover, Table 2 presents information detailing 40 differential expressed genes with the same expression trends in the transcriptome and MSLQP.

**Table 2:** The information detailing the 40 differential expressed genes with the same expressional trend in transcriptome and mass spectrometry-based label-free quantitative proteomics.

Protein	Gene	Protein_log2foldchange	Gene_log2foldchange	Type
P35610	ENSG00000057252	−0.69598	−0.9622	DEPs_DEGs
P27701	ENSG00000085117	−0.45572	−1.3729	DEPs_DEGs
Q07812	ENSG00000087088	−0.88497	−0.5869	DEPs_DEGs
Q9UBR2	ENSG00000101160	−0.46182	−0.7002	DEPs_DEGs
Q9UH62	ENSG00000102401	−0.62812	−0.7119	DEPs_DEGs
Q08380	ENSG00000108679	−0.74042	−2.252	DEPs_DEGs
Q13162	ENSG00000123131	−0.58827	−1.4554	DEPs_DEGs
Q9BVA1	ENSG00000137285	−0.54132	−0.9956	DEPs_DEGs
P05386	ENSG00000137818	−1.80749	−0.7395	DEPs_DEGs
P29373	ENSG00000143320	−1.0322	−3.2821	DEPs_DEGs
P05109	ENSG00000143546	−2.49366	−2.2156	DEPs_DEGs
Q9BQB6	ENSG00000167397	−1.34174	−1.9301	DEPs_DEGs
P45877	ENSG00000168938	−3.18924	−1.1132	DEPs_DEGs
Q6UWI2	ENSG00000169116	−0.77575	−0.9256	DEPs_DEGs
P14770	ENSG00000169704	−3.93356	−2.061	DEPs_DEGs
P16278	ENSG00000170266	−0.64759	−0.7117	DEPs_DEGs
Q9UHL4	ENSG00000176978	−0.69681	−1.1755	DEPs_DEGs
Q53GA4	ENSG00000181649	−0.89176	−0.9465	DEPs_DEGs
P32322	ENSG00000183010	−2.17876	−2.4351	DEPs_DEGs
Q6P988	ENSG00000185269	−3.51126	−4.6513	DEPs_DEGs
Q8IYD1	ENSG00000189369	−1.99356	−1.4963	DEPs_DEGs
Q99829	ENSG00000214078	−1.13544	−0.6995	DEPs_DEGs
Q5VT79	ENSG00000264230	−1.4677	−1.0327	DEPs_DEGs
O43491	ENSG00000079819	0.4068	0.9248	DEPs_DEGs
Q9Y6E0	ENSG00000102572	0.430783	1.3518	DEPs_DEGs
Q8NEV8	ENSG00000110723	0.983687	0.9765	DEPs_DEGs
O75533	ENSG00000115524	0.422799	0.8417	DEPs_DEGs
O15229	ENSG00000117009	0.761241	1.3381	DEPs_DEGs
P14902	ENSG00000131203	0.732334	1.3823	DEPs_DEGs
Q13136	ENSG00000131626	0.450986	0.9663	DEPs_DEGs
Q9UBP9	ENSG00000144366	0.422473	0.7059	DEPs_DEGs
Q86SQ0	ENSG00000144824	0.686685	0.7838	DEPs_DEGs
Q7Z5G4	ENSG00000147533	0.755982	1.4745	DEPs_DEGs
Q8TDX7	ENSG00000151414	0.532134	1.0977	DEPs_DEGs
Q01831	ENSG00000154767	1.123603	1.3083	DEPs_DEGs
Q6UXV4	ENSG00000155008	0.858467	0.9169	DEPs_DEGs
Q6WKZ4	ENSG00000156675	0.514716	1.1451	DEPs_DEGs
Q6UXG3	ENSG00000161649	1.501854	1.029	DEPs_DEGs
P29474	ENSG00000164867	1.215975	1.3914	DEPs_DEGs
Q9BX67	ENSG00000166086	0.412658	1.8249	DEPs_DEGs

Gene Ontology and Kyoto Encyclopedia of Genes and Genomes were used to analyse the function and signalling pathways of the correlated genes in the transcriptome and MSLQP.

**Fig. 8:**
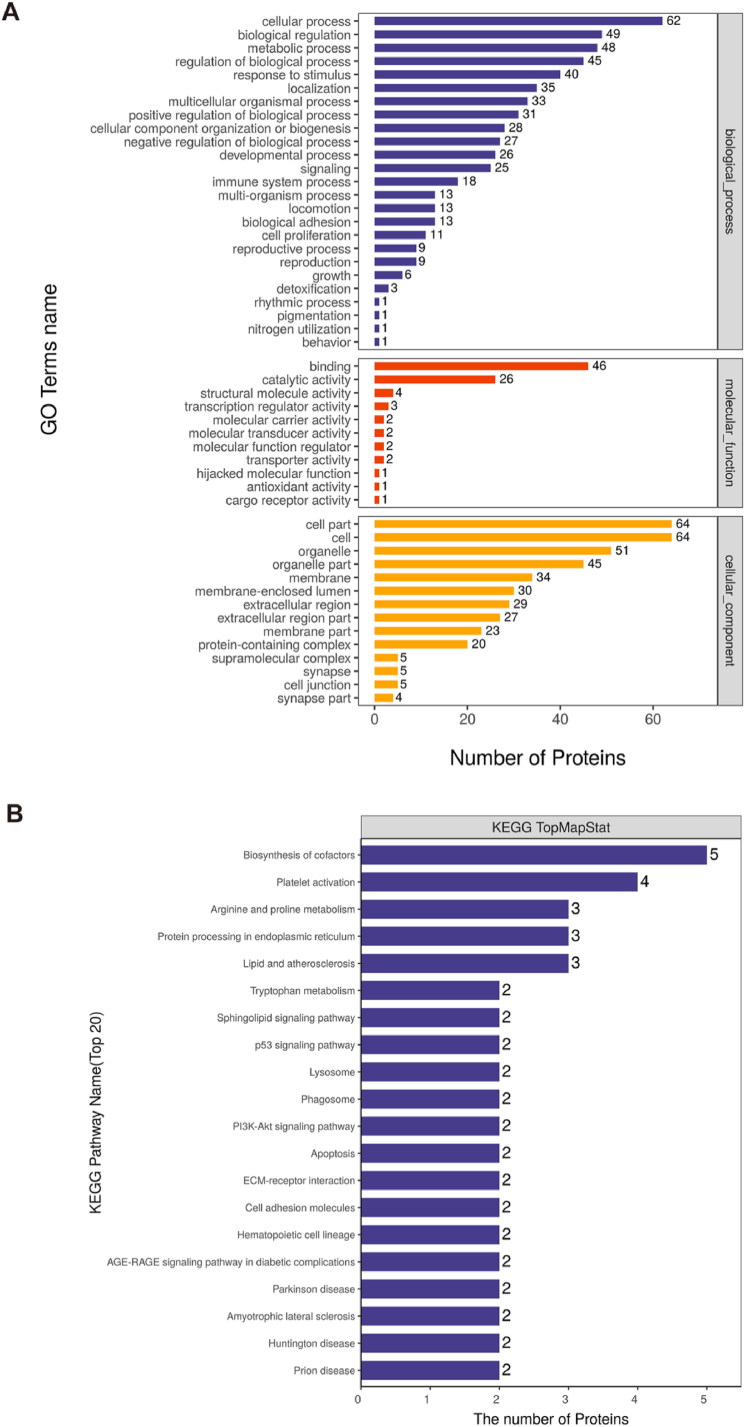
Gene Ontology and Kyoto Encyclopedia of Genes and Genomes were used to analyse the function and signalling pathways of the correlated genes in transcriptome and mass spectrometry-based label-free quantitative proteomics. (A) GO secondary classification of proteins. Each column represents a secondary classification of GO, and the three colours represent three major categories. Blue represents biological processes, orange represents molecular functions, and yellow represents cell components. The left ordinate represents the number of differential protein entries in the secondary classification, and the right ordinate represents the percentage of entries in the total differential protein number in the correlated genes. (B) Kyoto Encyclopedia of Genes and Genomes analysis of the top 20 pathways with the most significant numbers of differentially expressed proteins. The abscissa represents the name of the KEGG pathway, and the ordinate represents the number of differential proteins corresponding to the KEGG pathway. From top to bottom, the number of differentially expressed proteins in the association is ranked from high to low.

Furthermore, the functions and pathways of these genes were analysed. Fig. 8A and Table S6 presented the BP terms that include cellular process, response to stimulus, biological regulation, localization, multicellular organismal process, cellular component organization or biogenesis, developmental process, signalling, immune system process, multi-organism process, locomotion, biological adhesion, reproductive process, cell proliferation, detoxification, reproduction, growth, rhythmic process, nitrogen utilisation, pigmentation, and behavior. MF terms included binding, structural molecule activity, transcription regulator activity, catalytic activity, molecular carrier activity, molecular transducer activity, molecular function regulator, transporter activity, hijacked molecular function, antioxidant activity, and cargo receptor activity. The CC terms included cell, cell part, membrane, membrane-enclosed lumen, organelle, extracellular region part, membrane part, protein-containing complex, extracellular region, supramolecular complex, synapse, cell junction, and synapses part. The top 20 KEGG terms included the biosynthesis of cofactors, platelet activation, arginine and proline metabolism, molecules, protein processing in the endoplasmic reticulum, tryptophan metabolism, sphingolipid signalling pathway, p53 signalling pathway, ECM-receptor interaction, lysosome, phagosome, PI3K-AKT signalling pathway, apoptosis, cell adhesion, and haematopoietic cell lineage (Fig. 8B and Table S7).

### 3.8 Overexpression of RBM15 inhibited CD82 expression and its potential regulation mechanism

Table 2 indicated that the differentially expressed genes exhibited increased or decreased expression in both the PE and NC placental groups according to both mass spectrometry and transcriptome analyses. Through a literature review, we identified two genes (Q6P988 and P27701) that were related to trophoblast function, and the literature indicated that their expression was consistent with the results of our sequencing [[19], [20], [21], [22]]. Therefore, we hypothesized that these two factors might act downstream of RBM15. Q6P988, also termed NOTUM (palmitoleoyl-protein carboxylesterase) and a member of the α/β hydrolase family, belonged to the deacylated extracellular protein in the Wnt signalling pathway. Previous work showed that decreased NOTUM expression activated the Wnt pathway and inhibited stem cell differentiation of human trophoblast cells, ultimately resulting in PE and intrauterine growth restriction [19]. P27701, also known as CD82 (cluster of differentiation 82), belongs to the p53 signalling pathway. Studies have demonstrated that CD82 is a critical gene in the development of human trophoblasts and play a vital role in regulating the normal invasion and migration of trophoblast cells [[20], [21], [22]]. However, the roles of NOTUM and CD82 in the context of m^6^A modification during preeclampsia pathogenesis remain unclear.

**Fig. 9:**
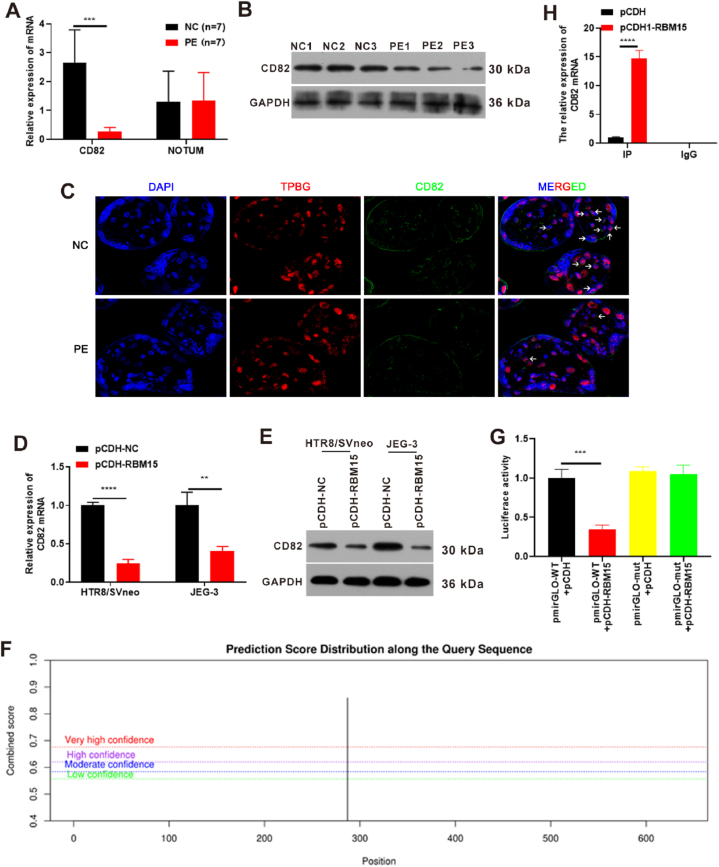
Overexpression of RBM15 inhibited CD82 expression and its potential regulation mechanism. (A) qPCR was used to detect the expression levels of NOTUM and CD82 in the placental NC and PE groups. N (biological replicates) = 7 each group. (B–C) Western blotting (B) and immunofluorescence (C) were used to detect CD82 abundance in the placenta of the NC and PE groups. TPGB is the markers of trophoblast cells. N (biological replicates) = 3 each group. (D) qPCR analysis of CD82 expression following RBM15 overexpression. The pCDH-RBM15 vector and its control vector, pCDH-NC, were transfected into HTR8/SVneo and JEG-3 cells for 24 h. (E) Western blotting was used to detect CD82 abundance after RBM15 overexpression. pCDH-RBM15 vector and its control vector pCDH-NC were transfected into cells for 48 h. (F) SRAMP predicted the m^6^A modification site of CD82 mRNA 3'UTR. (G) Effect of overexpression of RBM15 on the stability of the CD82 3’UTR was detected by luciferase activity. pmirGLO-CD82 3′UTR WT (pmirGLO-WT) or pmirGLO-CD82 3′UTR mut (pmirGLO-mut) vector and pCDH-RBM15 (or pCDH) were co-transfected into 293T cells for 48 h. (H) YTHDF2-RIP detection was used to evaluate the binding ability between YTHDF2 and the CD82 3′UTR. pCDH or pCDH-RBM15 were transfected into HTR8/SVneo for 48 h **p value less than 0.01, ***p value less than 0.001, ****p value less than 0.0001. PE, pre-eclampsia; NC, normal pregnancy placenta; CD82, cluster of differentiation 82; NOTUM, palmitoleoyl-protein carboxylesterase; RBM15, RNA-binding protein 15; GAPDH, glyceraldehyde-3-phosphate dehydrogenase; pCDH-NC, vector.

NOTUM and CD82 expression was detected in seven placenta samples from PE group and seven placenta samples from NC group. The results indicated that CD82 mRNA expression was increased in the PE group relative to that in the NC group, but NOTUM mRNA expression was not significantly different between two groups (Fig. 9A). Additionally, CD82 protein expression was lower in the PE group than it was in the NC group (Fig. 9B). Base on previous studies, trophoblast glycoprotein (TPGB) is strongly expressed throughout pregnancy in placental trophoblast cells and locates on the cell membrane [[23], [24], [25]]. Therefore, TPGB can be the markers of trophoblast cells. From the immunofluorescence results, CD82 protein expression was also lower in the PE group than it was in the NC group (Fig. 9C). To verify if RBM15 regulates CD82 expression, the pCDH-RBM15 vector and its control vector (pCDH-NC) were transfected into HTR8/SVneo and JEG-3 cells for 24 h (for qPCR detection) or 48 h (for Western blot analysis). The qPCR results revealed that CD82 expression was decreased to 50 % or greater after RBM15 overexpression (Fig. 9D). Western blot analysis confirmed the expression trend of the protein abundance of CD82 and was in agreement with the qPCR results (Fig. 9E). These results confirmed that RBM15 regulates CD82 expression. Then, SRAMP was used to predict the m^6^A modification site of CD82 mRNA 3'UTR, and the results indicated that there was a very high confidence position in CD82 mRNA 3'UTR (Fig. 9F). Based on this prediction, pmirGLO-CD82 3'UTR WT (pmirGLO-WT) and pmirGLO-CD82 3'UTR mut (pmirGLO-mut) vectors were constructed, and this was followed by luciferase activity testing. The results revealed that the pmirGLO-WT + pCDH-RBM15 group exhibited lower luciferase activity compared to that of the pmirGLO-WT + pCDH group, thus indicating that overexpression of RBM15 inhibited the stability of CD82 3'UTR (Fig. 9G). Moreover, the YTHDF2-RIP experiment demonstrated that CD82 expression was promoted in pCDH-RBM15 group compared to that in the pCDH group, thus indicating that the binding ability of YTHDF2 to CD82 3'UTR was increased after overexpression of RBM15 (Fig. 9H). These results confirmed the potential mechanism where overexpression of RBM15 inhibited CD82 expression. Additionally, overexpression of RBM15 increased the binding ability between YTHDF2 and the CD82 3'UTR to degrade CD82, thereby reducing CD82 expression.

### 3.9 RBM15 participated in the migration and invasion of trophoblast cells through the regulation of CD82

**Fig. 10:**
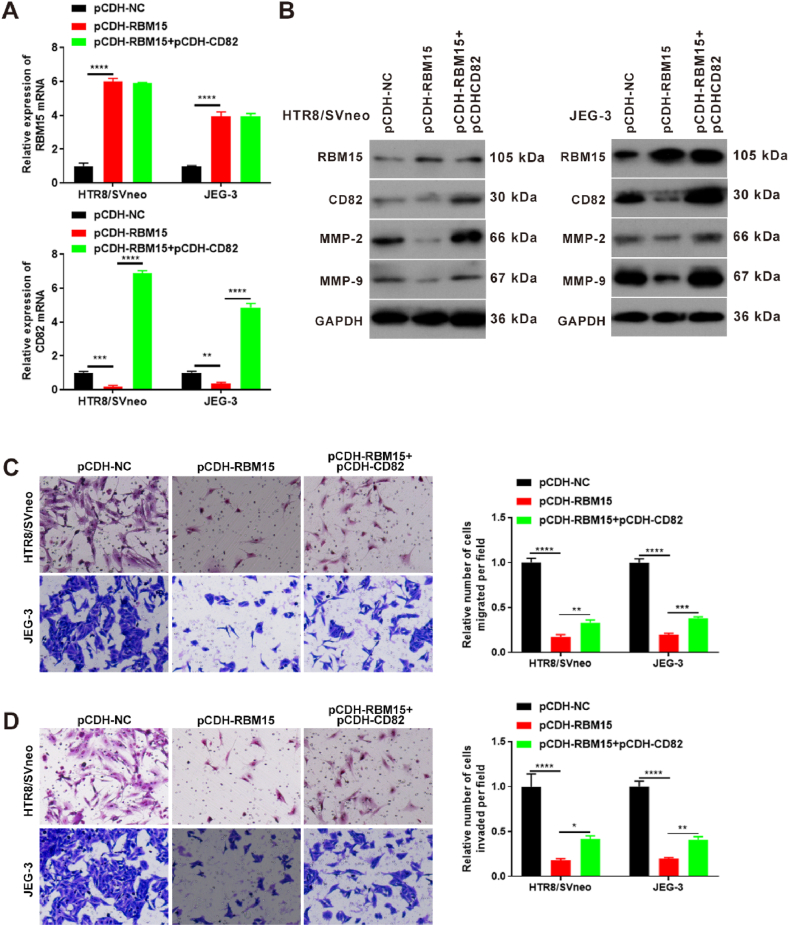
RBM15 participates in the effect of trophoblast cells via regulation CD82 in HTR8/SVneo and JEG-3 cells. (A) qPCR was used to detect the expression of RBM15 and CD82 after overexpression of RBM15 and CD82. pCDH, pCDH-RBM15, or pCDH-CD82 were transfected into cells for 24 h. (B) Western blotting was used to detect the abundance of RBM15, CD82, MMP-2, and MMP-9 after the overexpression of RBM15 and CD82. Cells were transfected with pCDH, pCDH-RBM15, or pCDH-CD82 for 48 h. (C) Transwell assays were used to detect changes in cell migration after overexpression of RBM15 and CD82. Cells were transfected with pCDH, pCDH-RBM15, or pCDH-CD82 for 48 h. (D) Transwell assays were used to detect changes in cell invasion after overexpression of RBM15 and CD8. pCDH, pCDH-RBM15, or pCDH-CD82 were transfected into cells for 48 h *p value less than 0.05; **p value less than 0.01; ***p value less than 0.001; ****p value less than 0.0001. RBM15, RNA-binding protein 15; MMP-2, matrix metallopeptidase 2; MMP-9, matrix metallopeptidase 9; GAPDH, glyceraldehyde-3-phosphate dehydrogenase; pCDH-NC, pCDH vector; CD82, cluster of differentiation 82.

To verify if RBM15 participates in trophoblast cell function via regulation of CD82, a pCDH-CD82 vector was constructed. The qPCR and western blotting results revealed that RBM15 expression was promoted and CD82 expression was inhibited after overexpression of RBM15. However, CD82 expression was reversed by CD82 overexpression but not by RBM15 mRNA and protein expression (Fig. 10A and B). Furthermore, the migration and invasion results demonstrated that the ability of migration and invasion was decreased to 20 % by overexpression of RBM15. Furthermore, overexpression of CD82 partially rescued the migration and invasion ability (Fig. 10C and D). Additionally, the levels of MMP-2 and MMP-9 were reduced after RBM15 overexpression, and this was reversed by CD82 overexpression (Fig. 10B). These results indicated that RBM15 participates in trophoblast cell function via the regulation of CD82.

## 4 Discussion

PE has been documented for more than 2,400 years and is a common disorder in pregnant women [26]. As the pathophysiology becomes clearer, biochemical marker assays are being developed to improve diagnosis and prediction, although there are still no effective treatment strategies [2]. Therefore, it is imperative to investigate the pathological mechanisms underlying PE. In this study, we identified 16 m^6^A modification-related proteins in the placenta. We focused on the top 3 differentially expressed proteins (G3BP1, RBM15, and YTHDF2) in the PE and NC groups. Only RBM15 abundance in PE is increased, and the expression trend was the same as that observed in the proteomics analysis. After RBM15 overexpression, total m^6^A modification was enhanced, but cell migration and invasion abilities were reduced. In total, 2,751 mRNAs were significantly differentially expressed between the PE and NC groups. Only 40 mRNAs exhibited the same expression trend as that of the proteomics analysis. P27701 (also named CD82) that participates in the development of human trophoblasts was inhibited by RBM15 overexpression. CD82 expression was lower in the PE group than it was in the NC group. Furthermore, the function of overexpressed RBM15 was rescued by overexpressed CD82. We also determined that the potential mechanism was that overexpression of RBM15 increased the binding ability between YTHDF2 and CD82 3′UTR, thus decreasing the CD82 expression. For the first time, our research combined proteomics and transcriptomics experiments to conduct bioinformatics analysis, ultimately providing a “panoramic view” of the expression spectrum for the potential pathological process of PE that provided a direction for further research. Simultaneously, the combined analysis of proteomics and genomics provided favourable information for m^6^A modification. We also observed that the m^6^A regulatory gene RBM15 can participate in the process of PE by affecting the binding ability of YTHDF2 to the CD82 3′UTR, thereby reducing the expression of CD82.

RBM15 acts as a “writer” of m^6^A modification in multiple diseases [27,28]. For example, Overexpression of RBM15 increased laryngeal squamous cell carcinoma cell proliferation, migration, invasion, and apoptosis by regulating TMBIM6 stability in an IGF2BP3-dependent manner [29]. Additionally, previous studies reported that RBM15 is involved in cell fate decisions such as megakaryopoiesis, haematopoietic stem cell modulation, and liver and brain development [[30], [31], [32]]. For example, knocking out RBM15 in a differentiated liver using CRISPR/Cas9 inhibited hepatic maturation without affecting hepatoblast specification, differentiation, proliferation, or apoptosis of hepatocytes [32]. RBM15 modulated the function of BAF155 through RNA methylation in the cortex, thus resulting in development [33]. RBM15 plays an essential role in metabolic diseases. For example, RBM15 was observed to hinder insulin sensitivity and promote insulin resistance through m6A-mediated epigenetic suppression of CLDN4 during the development of gestational diabetes mellitus [34]. More importantly, a previous *in vivo* study determined that the knockdown of RBM15 led to defects in the morphology of placental vascular branches within the syncytiotrophoblast and spongy trophoblast cell layers that play an essential role in placental insufficiency and cardiac malformation [35]. However, the mechanism of RBM15 action in the placenta remains unclear. This study observed that RBM15 expression was promoted in PE and confirmed the importance of RBM15 in trophoblast migration and invasion *in vitro*. Overexpression of RBM15 also inhibited trophoblast migration and invasion. Furthermore, we also revealed the potential mechanism of RBM15 on trophoblast function, where overexpression of RBM15 reduced CD82 expression by increasing the binding ability of YTHDF2 and CD82 3′UTR.

CD82 is a glycoprotein and a member of the tetraspanin family. CD82 is located on the cell membrane and possesses four transmembrane domains that include a large and a small extracellular domain and cytoplasmic N- and C-termini. The extracellular domain of CD82 contains N-glycosylated asparagine residues that are divided into constant and variable regions [36]. The constant region contains α-helices, variable region, most of the known protein-protein interaction sites, and cysteine residues that form intramolecular disulfide bonds [37]. Cysteine residues located in the cytoplasmic domain that is adjacent to the membrane undergo palmitoylation. Therefore, CD82 plays an essential role in various biological activities, including cell motility, migration, invasion, metastasis, and membrane protrusion [[38], [39], [40]]. A previous study confirmed the function of CD82. For example, N-glycosylation at the Asn157 of CD82 decreased metastases in lung cancer and adhesion in colorectal cancer via down-regulating Wnt/β-catenin activation [41]. Upon the activation of anti-inflammatory mechanisms, CD82 exerted inhibitory effects on the migration of neutrophils and macrophages by altering cell morphology and reversing the expression or metabolism of damage-related genes [42]. In retinal pigment epithelial cells, CD82 interacted with TGFRs and integrins to suppress epithelial-mesenchymal transition by decreasing the activation of the Smad-dependent pathway [43]. Additionally, a previous study indicated strong expression levels of CD82 in decidual cells, whereas negative expression levels of CD82 were observed in trophoblast cells. CD82 may also participated in the communication and invasion regulation of trophoblast cells [44]. Two additional studies confirmed that trophoblast cell-derived CXCL12 controlled the excessive invasion of trophoblasts by enhancing CD82 expression in decidual stromal cells [22,45]. Furthermore, Zhang et al. reported that CD82 influenced the migration and invasion of HTR8/SVneo cells and villous explants. These phenomena may be associated with MMP expression [46]. The present study also confirmed that overexpression of CD82 decreased the inhibitory effect of RBM15 overexpression on gene expression (CD82, MMP-2, and MMP-9) and the ability of migration and invasion of trophoblast cells. Previous study showed that low expression of CD82 promotes the migration and invasion abilities of HTR8/SVneo cell [46]. Gene regulation is influenced by multi-factors rather than single factor. Overexpression of RBM15 and CD82 may have interaction effect, inducing different regulatory effect. Further study is needed to investigate the mechanism of this regulation.

However, this study had some limitations. First, our study lacked *in vivo* experiments to confirm this conclusion. Second, the potential signalling pathways regulated by RBM15/CD82 require verification. Third, additional clinical samples should be collected to confirm RBM15 and CD82 expression. Therefore, future studies should focus on these experiments.

## 5 Conclusions

We determined that RBM15, an m^6^A regulator, was upregulated in PE. RBM15 overexpression increased total m^6^A modification; however, it inhibited the migratory and invasive capabilities of trophoblast cells and CD82 expression. Furthermore, CD82 overexpression reversed the function of the overexpression of RBM15 on trophoblast cells. Mechanically, overexpression of RBM15 enhanced the binding ability between YTHDF2 and the CD82 3′UTR, thus leading to a decreased CD82 expression. Our study provides a new theoretical basis for methylation modifications during PE treatment.

## Ethics approval and consent to participate

This study was conducted in accordance with the 1964 Declaration of Helsinki and was approved by the Ethics Committee of the Third Affiliated Hospital of Sun Yat-sen University (ethics number II2023-063-01). All participants/patients (or their proxies/legal guardians) provided informed consent to participate in the study. All participants/patients (or their proxies/legal guardians) provided informed consent for the publication of their anonymised case details.

## Funding

This study was supported by the 10.13039/501100003453Natural Science Foundation of Guangdong Province (grant number: 2021A1515011441) and the 10.13039/501100001809National Natural Science Foundation of China (grant number: 82070606). The authors declare no conflict of interest.

## Data availability statement

Data will be made available on request.

## CRediT authorship contribution statement

**Guangning You:** Writing – review & editing, Writing – original draft, Validation, Software, Methodology, Investigation, Formal analysis, Data curation, Conceptualization. **Zhe Li:** Writing – review & editing, Supervision. **Ling Li:** Writing – review & editing. **Chengfang Xu:** Writing – review & editing, Supervision.

## Declaration of competing interest

The authors declare that they have no known competing financial interests or personal relationships that could have appeared to influence the work reported in this paper.
