# Supplementary material for: Overexpression of RBM15 modulated the effect of trophoblast cells by promoting the binding ability between YTHDF2 and the CD82 3′UTR to decrease the expression of CD82
Source: Heliyon. 2024 May 3;10(9):e30702. doi: 10.1016/j.heliyon.2024.e30702 (PMC11098837; doi:10.1016/j.heliyon.2024.e30702)
Supplement: Multimedia component 2 [file mmc2.pdf]

RAW DATA

Figure 3A

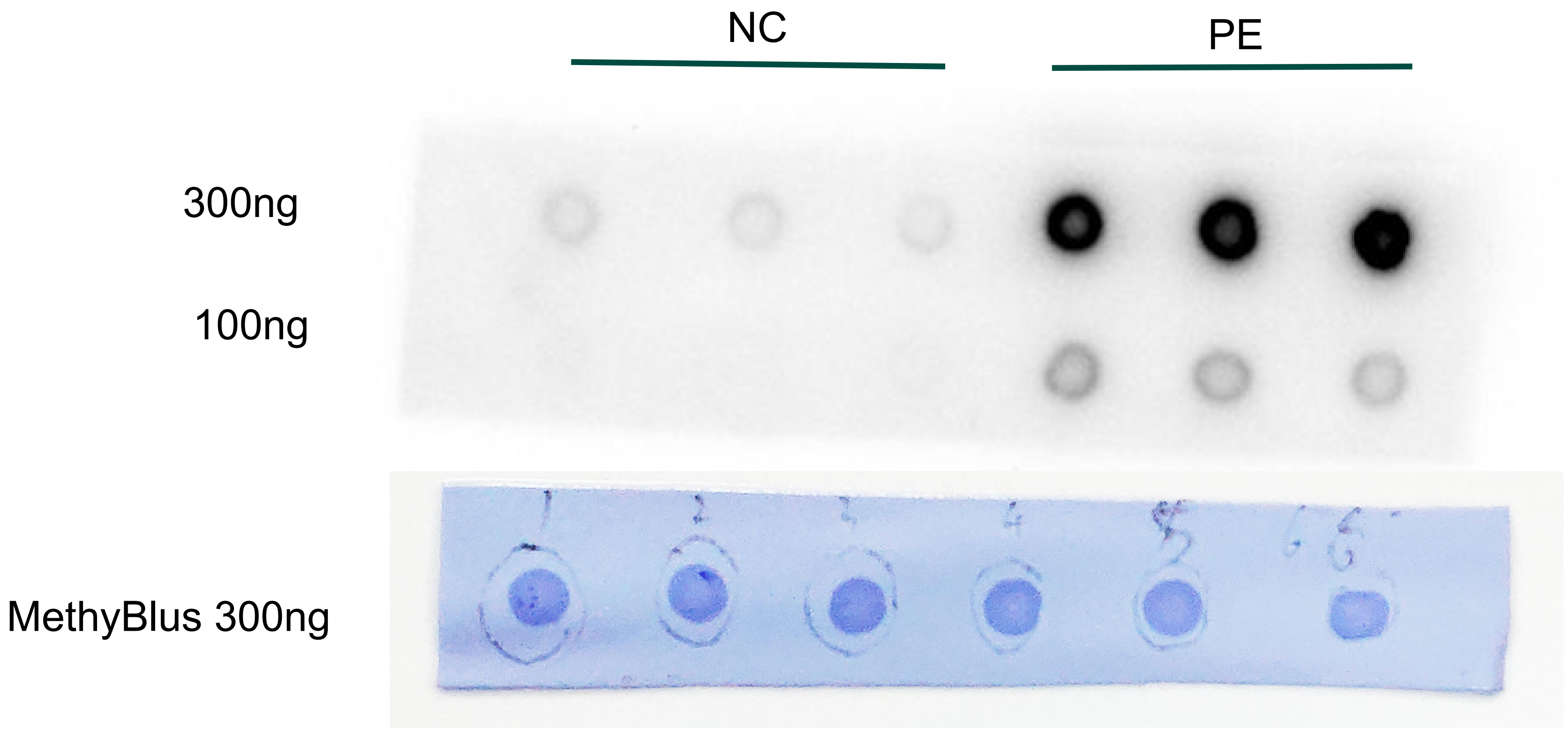

name	brand	CAT	dilution	species
anti-m6A antibody	proteintech	68055-1-Ig	1:2000	MS
Goat anti-Mouse IgG (H&L)-HRP	proteintech	PR30012	1:2000	

Figure 3C

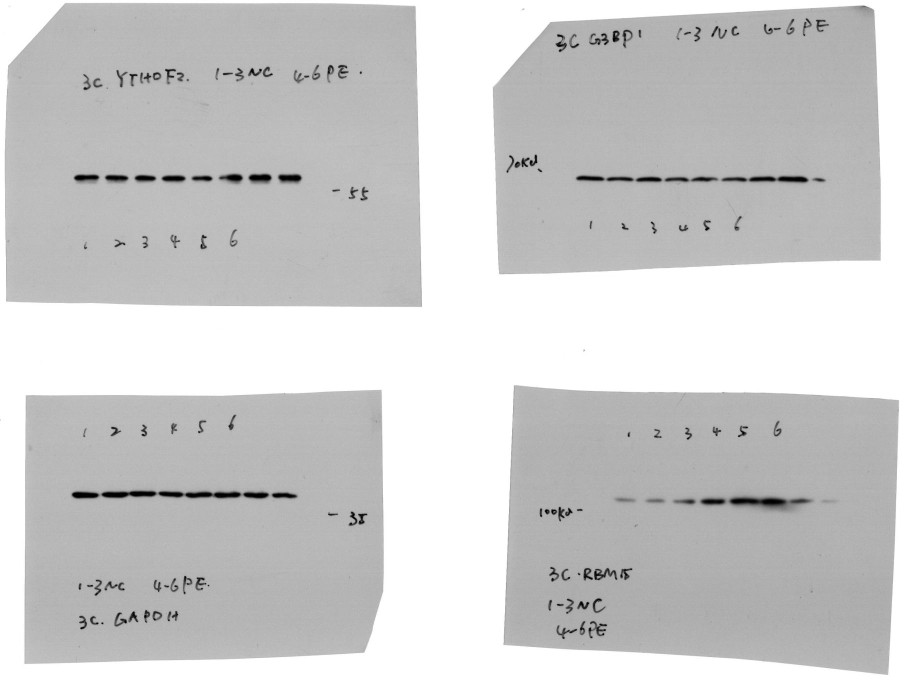

name	brand	CAT	Mol. wt.	dilution	species
RBM15 Polyclonal antibody	proteintech	10587-1-AP	100-107 kDa	1:4000	RB
G3BP1 Polyclonal antibody	proteintech	13057-2-AP	68 kDa	1:8000	RB
YTHDF2 Polyclonal antibody	proteintech	24744-1-AP	62 kDa	1:5000	RB
GAPDH Polyclonal antibody	proteintech	10494-1-AP	36 kDa	1:20000	RB
Goat anti-Rabbit IgG (H&L)-HRP	proteintech	PR30011	NO	1:20000	

Figure 4B

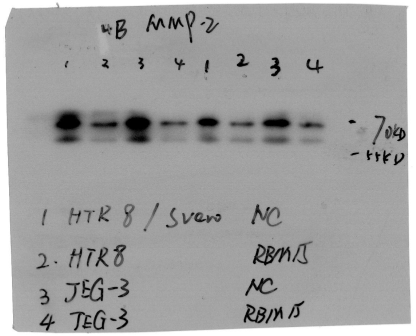

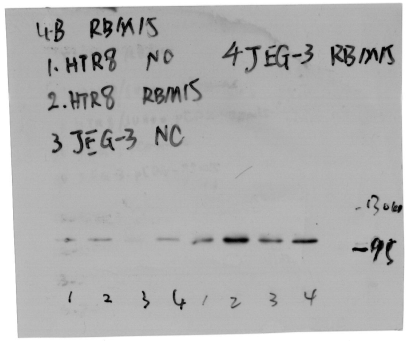

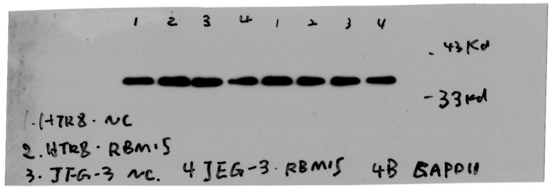

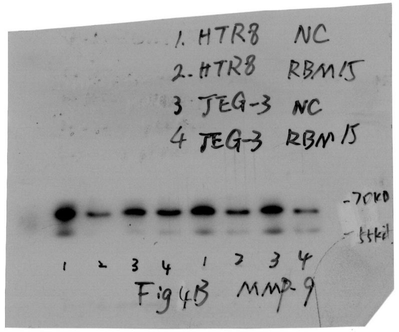

name	brand	CAT	Mol. wt.	dilution	species
RBM15 Polyclonal antibody	proteintech	10587-1-AP	100-107 kDa	1:4000	RB
MMP2 Polyclonal antibody	proteintech	10373-2-AP	62-72 kDa	1:500	RB
MMP9 (N-terminal) Polyclonal antibody	proteintech	10375-2-AP	67 kDa	1:1000	RB
GAPDH Polyclonal antibody	proteintech	10494-1-AP	36 kDa	1:20000	RB
Goat anti-Rabbit IgG (H&L)-HRP	proteintech	PR30011	NO	1:20000	

Figure 4C

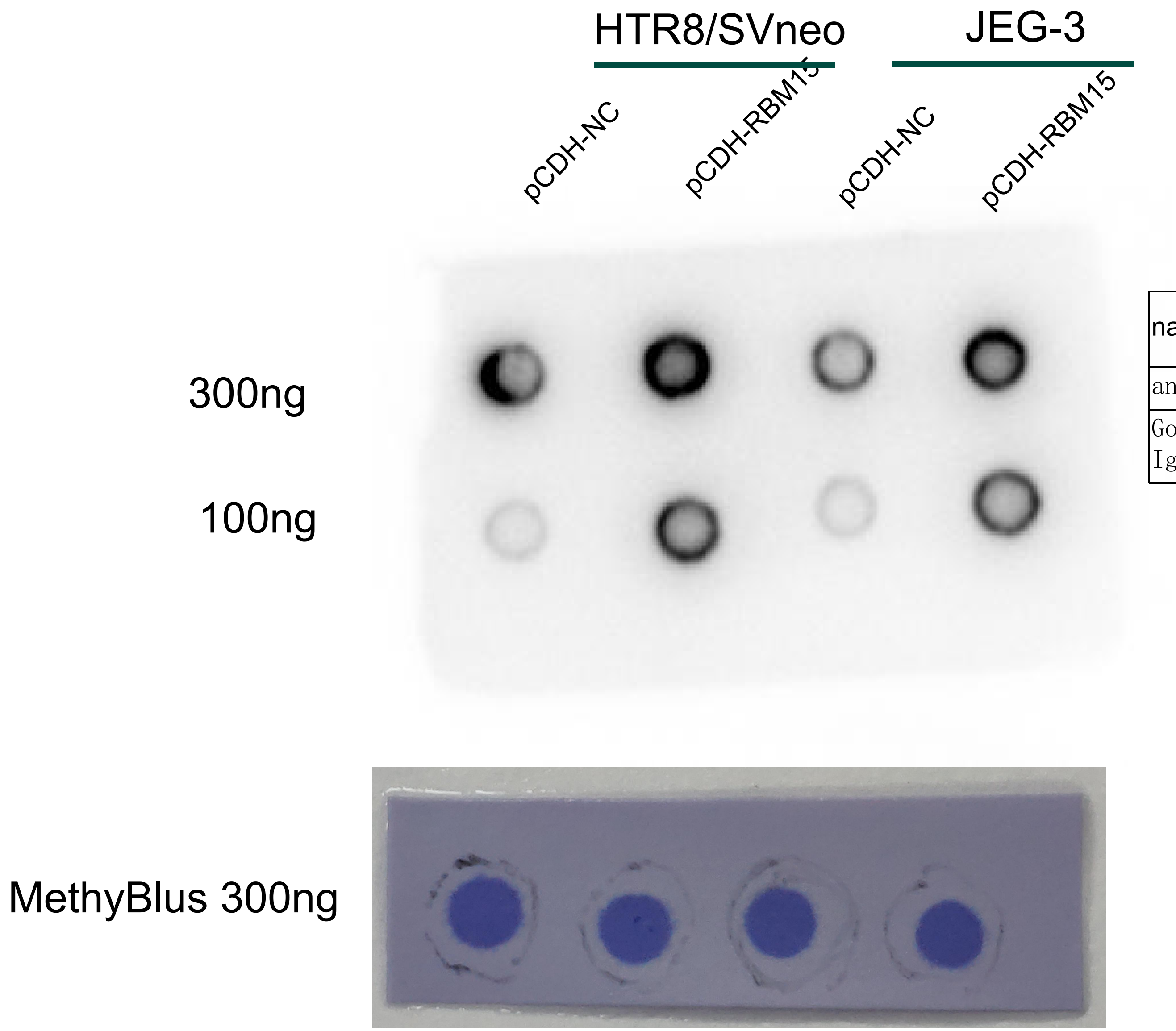

name	brand	CAT	dilution	species
anti-m6A antibody	proteintech	68055-1-Ig	1:2000	MS
Goat anti-Mouse IgG (H&L)-HRP	proteintech	PR30012	1:2000	

Figure 9B

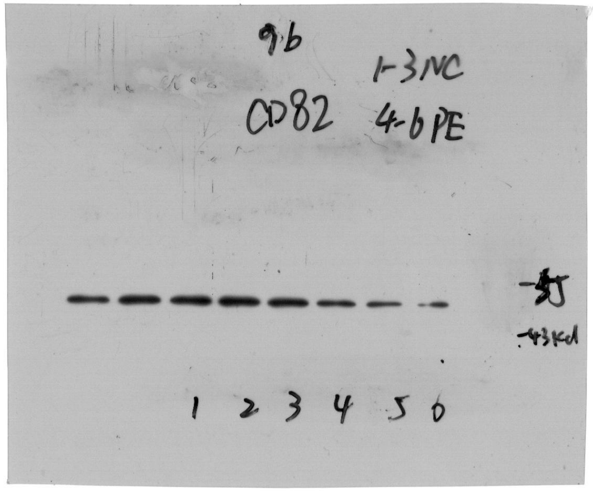

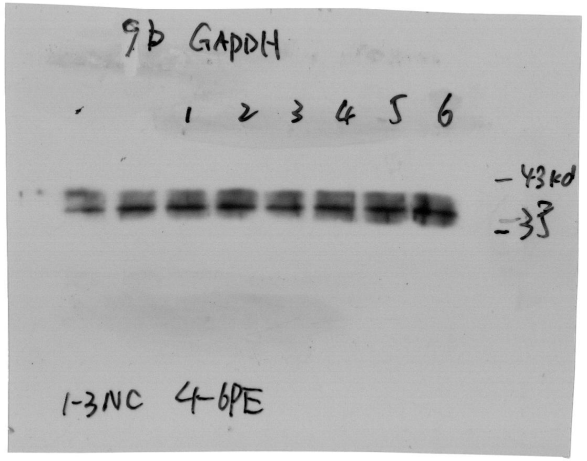

name	brand	CAT	Mol. wt.	dilution	species
GAPDH Polyclonal antibody	proteintech	10494-1-AP	36 kDa	1:20000	RB
CD82 Monoclonal antibody	proteintech	66803-1-Ig	48 kDa	1:3000	MS
Goat anti-Mouse IgG (H&L)-HRP	proteintech	PR30012	NO	1:20000	
Goat anti-Rabbit IgG (H&L)-HRP	proteintech	PR30011	NO	1:20000	

Figure 9E

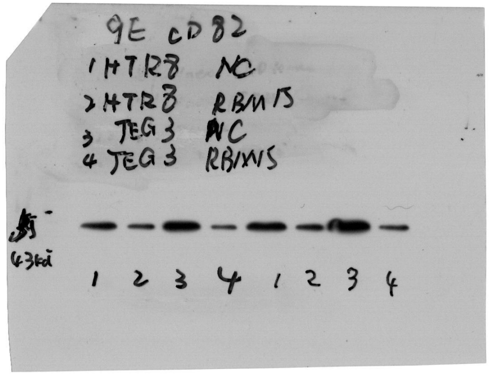

name	brand	CAT	Mol. wt.	dilution	species
GAPDH Polyclonal antibody	proteintech	10494-1-AP	36 kDa	1:20000	RB
CD82 Monoclonal antibody	proteintech	66803-1-Ig	48 kDa	1:3000	MS
Goat anti-Mouse IgG (H&L)-HRP	proteintech	PR30012	NO	1:20000	
Goat anti-Rabbit IgG (H&L)-HRP	proteintech	PR30011	NO	1:20000	

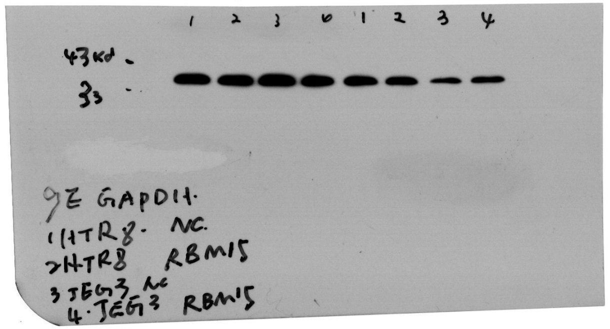

Figure 10B

HTR8/SVneo

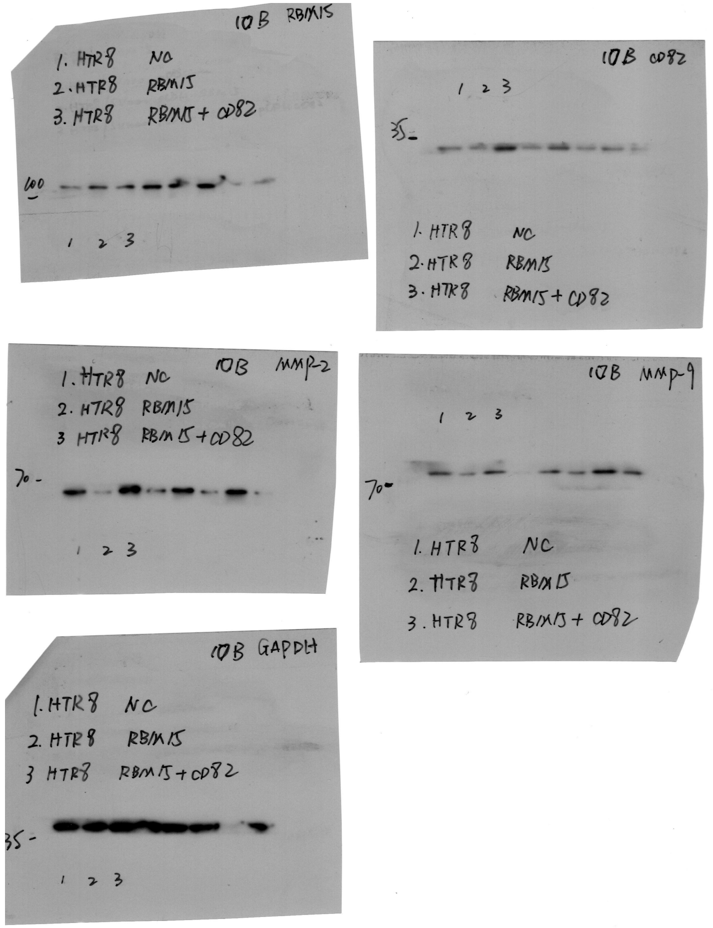

JEG-3

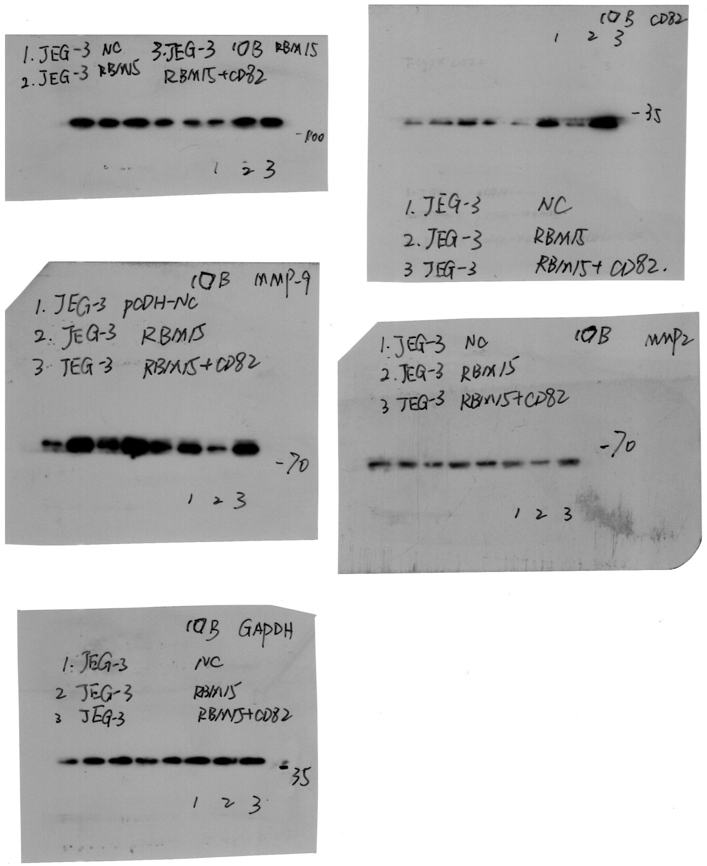

name	brand	CAT	Mol. wt.	dilution	species
RBM15 Polyclonal antibody	proteintech	10587-1-AP	100-107 kDa	1:4000	RB
MMP2 Polyclonal antibody	proteintech	10373-2-AP	62-72 kDa	1:500	RB
MMP9 (N-terminal) Polyclonal antibody	proteintech	10375-2-AP	67 kDa	1:1000	RB
GAPDH Polyclonal antibody	proteintech	10494-1-AP	36 kDa	1:20000	RB
CD82 Monoclonal antibody	proteintech	66803-1-Ig	48 kDa	1:3000	MS
Goat anti-Mouse IgG (H&L)-HRP	proteintech	PR30012	NO	1:20000	
Goat anti-Rabbit IgG (H&L)-HRP	proteintech	PR30011	NO	1:20000	
